# Expression profiling reveals Spot 42 small RNA as a key regulator in the central metabolism of *Aliivibrio salmonicida*

**DOI:** 10.1186/1471-2164-13-37

**Published:** 2012-01-24

**Authors:** Geir Å Hansen, Rafi Ahmad, Erik Hjerde, Christopher G Fenton, Nils-Peder Willassen, Peik Haugen

**Affiliations:** 1 Department of chemistry, Faculty of science and technology, University of Tromsø, N-9037, Tromsø, Norway; 2 The Norwegian Structural Biology Centre, University of Tromsø, N-9037, Tromsø, Norway; 3 Institute of Clinical Medicine, University of Tromsø, N-9037 Tromsø, Norway

## Abstract

**Background:**

Spot 42 was discovered in *Escherichia coli *nearly 40 years ago as an abundant, small and unstable RNA. Its biological role has remained obscure until recently, and is today implicated in having broader roles in the central and secondary metabolism. Spot 42 is encoded by the *spf *gene. The gene is ubiquitous in the *Vibrionaceae *family of gamma-proteobacteria. One member of this family, *Aliivibrio salmonicida*, causes cold-water vibriosis in farmed Atlantic salmon. Its genome encodes Spot 42 with 84% identity to *E. coli *Spot 42.

**Results:**

We generated a *A. salmonicida spf *deletion mutant. We then used microarray and Northern blot analyses to monitor global effects on the transcriptome in order to provide insights into the biological roles of Spot 42 in this bacterium. In the presence of glucose, we found a surprisingly large number of ≥ 2X differentially expressed genes, and several major cellular processes were affected. A gene encoding a pirin-like protein showed an on/off expression pattern in the presence/absence of Spot 42, which suggests that Spot 42 plays a key regulatory role in the central metabolism by regulating the switch between fermentation and respiration. Interestingly, we discovered an sRNA named VSsrna24, which is encoded immediately downstream of *spf*. This new sRNA has an expression pattern opposite to that of Spot 42, and its expression is repressed by glucose.

**Conclusions:**

We hypothesize that Spot 42 plays a key role in the central metabolism, in part by regulating the pyruvat dehydrogenase enzyme complex via pirin.

## Background

Bacteria contain a class of regulatory non-coding (nc) RNAs that are transcribed in *trans *from distinct promoters [1]. They are typically between 50 and 200 nt in size [2] and have become known as bacterial small RNAs, or sRNAs. The majority of known sRNA genes are located in intergenic regions, but it is becoming increasingly evident that a relatively large number of RNAs are also being transcribed from within protein coding regions, but from the opposite strand (i.e., anti-sense RNAs). Even though their roles are still mostly unknown, it is likely that anti-sense RNAs also play important roles in gene regulation. *cis*-encoded anti-sense RNAs share extensive complementarity to their messenger RNA (mRNA) target, whereas *trans*-encoded RNAs typically show limited complementarity [1]. Although *cis*-encoded RNA regulators can be encoded from within their mRNA target, they are typically located in front of protein coding regions as part of the mRNA. Here they change the expression of the corresponding protein by binding small metabolites (i.e., riboswitches) [3].

sRNAs typically bind to the 5' end of mRNAs through short imperfect base-paring and induce degradation of itself and the target [1]. Other mechanisms, like direct interaction with proteins to modulate their activities or increase stability of mRNAs, also occur. Finding the function and/or mechanism of sRNAs can however be a daunting task, and the Spot 42 sRNA represents a striking example. It was first described as an unstable RNA species of 109 nt in *Escherichia coli *that accumulated under growth in the presence of glucose (i.e., when adenosine 3', 5'-cyclic monophosphate (cAMP) is low) [4]. During growth with a non-glucose carbon source (i.e., when cAMP concentrations are high), the Spot 42 concentrations were found to be significantly lower. Later experiments showed that over-expression of Spot 42 (~10 fold increase) resulted in impaired growth and lowered ability to adapt to shifts to richer media or shift from glucose to succinate as the carbon source [5]. Also, deletion of the gene that encodes Spot 42, i.e., *spf *(spot fourty-two), resulted in viable *spf *null mutants, which indicated that the Spot 42 RNA is non-essential [6]. The direct responsiveness of Spot 42 levels to glucose and cAMP is due to repression of *spf *by a cAMP-CRP (cAMP-receptor protein) complex [7,8]. It was unclear for some years if the function of Spot 42 was mediated through the 109 nt RNA itself or through a 14 amino acids long peptide that is hypothetically encoded from within the sRNA. This controversy was settled by Rice *et al. *[9]. They used a filter binding assay and other methods to show that Spot 42 bound very inefficiently and nonproductive to purified 70S ribosomes, and concluded that Spot 42 function is mediated by the RNA itself. Later, the proximity of *spf *to *polA *(encodes DNA polymerase I) lead Dahlberg and co-workers to test whether the products of these genes could influence each other [7,8], and they found that reduction in Spot 42 levels, either by deletion of *spf *or by manipulating the growth conditions, both resulted in reduction in DNA pol A activity. The underlying mechanism remains however unknown. The first direct Spot 42 target was discovered by Møller *et al. *[10], who showed that the sRNA can bind specifically by base pairing with a short complementary region of the translation initiation region of *galK *(encodes a galactokinase), which is the third gene of the galactose operon (*galETKM*). The Spot 42-binding region overlaps with the *galK *Shine-Dalgarno region, thereby blocking ribosome binding. Spot 42 is therefore responsible for a discoordinate regulation of the *gal *operon. Finally, in a recent work Beisel and Storz [11] demonstrated with microarray analysis and reporter fusions that Spot 42 plays a broader role in metabolism by regulating at least fourteen operons dominated by genes involved in uptake and catabolism of non-favored carbon sources. Several of these operons are regulated by both Spot 42 and CRP, and these two regulators can therefore be considered as participating in a feed-forward loop.

In this study, we used our model bacterium *Aliivibrio salmonicida*, which belongs to the *Vibrionaceae *family of gamma-proteobacteria, to further address the biological roles of Spot 42. When we started the project, *galK *was the only known Spot 42 target. Interestingly, we found that *A. salmonicida *naturally lacks the *gal *operon, but contains a highly conserved *spf *gene, and we therefore considered this bacterium as an excellent model for studying Spot 42 roles other than in *galK *regulation. Even though other Spot 42 targets have since been proposed [11], our study is still valid, and our goal remains the same, i.e., to identify all biological roles of Spot 42. We generated an *A. salmonicida spf *deletion mutant and used microarray and Northern blot analyses to find more clues to its function. Deletion of *spf *has a surprisingly large effect on the transcriptome, with the most dramatic effect on expression of a pirin-like protein gene. In the process, we also discovered a neighboring sRNA, named VSsrna24, which is encoded from the same intergenic region (IGR) and 262 nt downstream of *spf*. Interestingly, this RNA is expressed in a pattern opposite to that of Spot 42, and its expression is repressed by glucose.

## Results and Discussion

### *spf *is highly conserved in *Vibrionaceae *and is located upstream of a previously unrecognized sRNA gene named *VSsrna24*

We first surveyed available genome sequences from the *Vibrionaceae *family and found that orthologs of *spf *are found in virtually all 76 currently available genomes. Available genomes include representatives of the *Vibrio*, *Aliivibrio*, *Photobacterium*, and *Grimontia *genera. Six *V. cholerae *genomes lack a recognizable *spf *gene, but we suspect that this is due to the poor quality of these genomes (i.e., the genome sequences consist of many small unfiltered contigs) or the lack of data. By using the *spf *sequence of *A. salmonicida *as the query in a Blastn search, the gene was also identified in fourteen gamma-proteobacteria genera (*Pantoea, Xenorhabdus, Salmonella, Citrobacter, Yersinia, Serratia, Edwardsiella, Dickeya, Photorhabdus, Escherichia, Enterobacter, Klebsiella, Rahnella *and *Shigella*) in the *Enterobacteriales *order.

**Figure 1:**
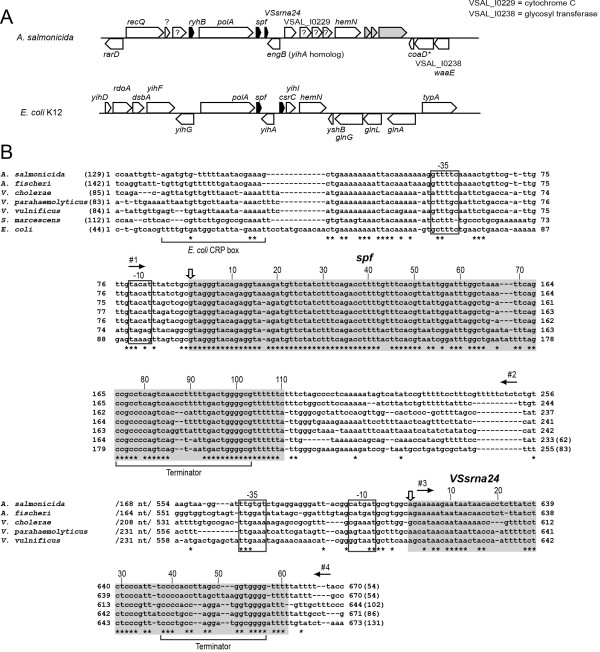
**Synteny comparison and sequence alignment of *spf *and *VSsrna24***. (A) The order of genes that flank *spf *and other sRNA genes (filled arrows) in *A. salmonicida *and *E. coli *are shown. Question marks indicate genes with unknown function. (B) Sequence alignment of *spf *and *VSsrna24 *from selected members of *Vibrionaceae*, *E. coli *and *Serratia marcescens*. The CRP binding site of *E. coli*, and -10 and -35 promoter regions in front of *spf *and *VSsrna24 *are based on knowledge from *E. coli *[7,8]. The vertical open arrows denote the 5' end of Spot 42 and *VSsrna24 *based on 5' RACE data on *A. salmonicida *presented in this work. The horizontal arrows (#1-4) denote areas deleted in the *spf *(#1-2) and *VSsrna24 *(#3-4) deletion strains. Numbers in parenthesizes denote numbers of nt to the nearest CDS. Asterisks indicate invariable positions.

Figure 1A shows the genomic location of *spf *and its flanking genes in *A. salmonicida *compared to the corresponding region in *E. coli*. In both genomes, *spf *is flanked by *polA *as the nearest upstream neighbor and *yihA *(or the *engB *homolog) as the nearest downstream protein coding gene. The genomic locus is also home to other important sRNA genes. In *A. salmonicida*, RyhB is encoded from the neighboring and upstream IGR [12]. Interestingly, *spf *is also neighbor to a putative sRNA gene (*VSsrna24*), which is located in the same IGR as *spf*. VSsrna24 was predicted in a global computer-based search for sRNAs that will be reported elsewhere. In *E. coli*, the IGR downstream of *spf *encodes the carbon storage regulator C (CsrC) sRNA that interacts directly with and regulates the activity of the carbon storage regulator A protein (CsrA) [13], which functions as a global post transcriptional regulator.

Figure 1B shows an alignment of Spot 42 and VSsrna24 from selected species of *Vibrionaceae*, and *Serratia marcescens *and *E. coli*. Spot 42 is found widespread among gamma-proteobacteria, whereas VSsrna24 is limited to *Vibrionaceae*, i.e., in *A. salmonicida*, *A. fischeri*, *V. cholera*, *V. furnissi*, *V. vulnificus*, *V. harveyi*, *V. parahaemolyticus *and *V. natriegens*. A 5' rapid amplification of cDNA ends (5' RACE) analysis was done to map the 5' end of the *A. salmonicida *Spot 42 and VSsrna24, and the result showed that the Spot 42 5' end is the same as for *E. coli *(indicated by vertical arrow in Figure 1B), and that VSsrna24 is approximately 60 nt in length. Ninety-one of the 109 nt in *E. coli *Spot 42 are invariable when compared to the selected sequences. The highly conserved nature and the wide distribution of *spf *in *Vibrionaceae *and other gamma-proteobacteria suggest that, as in *E. coli*, Spot 42 serves important cellular functions that might be common to this group of bacteria, regardless if the bacterium is a pathogen or not (although conservation is not always a sign of viability). Since 2002 and until recently, *galK *was the only known target of Spot 42 [10]. *A. salmonicida *naturally lacks the *gal *operon (including *galK*), but at the same time contains a highly conserved *spf *gene. At the onset of this project we therefore considered the bacterium as an ideal model for studying roles of Spot 42 other than regulation of *galK *translation. This is still valid even though other targets have been found [11]. In agreement with the lack of a *gal *operon, the bacterium is unable to utilize galactose when grown in a minimal medium optimized for *A. salmonicida*, with 44.4 mM galactose as essentially the only carbon source (data not shown). In other words, galactose has little or no effect on growth when compared to a control with no added sugar. In contrast, when 44.4 mM glucose is added as carbon source, the bacterium grows to high densities [i.e, typically to optical density 600 nm (OD_600 nm_) > 7].

Next, we examined the sequence upstream of *spf *for potential transcription factor binding sites to gain insights into regulation of *spf *transcription. Sequences that weakly resemble the *E. coli *CRP binding site, and -10 and -35 promoter regions were predicted upstream of *Vibrionaceae spf *sequences.

### Expression of Spot 42 and the adjacent VSsrna24 is highly dependent on glucose

It is established that expression of *E. coli *Spot 42 is repressed by the transcription factor CRP and cAMP [7]. Expression is also highly dependent on concentrations of available glucose since cAMP adenylyl cyclase (i.e., a cAMP-producing enzyme) is inhibited as a side-effect during glucose transport into the cell. As a result, cAMP levels are low when glucose is rich and is being transported into the cell for consumption, and vice versa.

**Figure 2:**
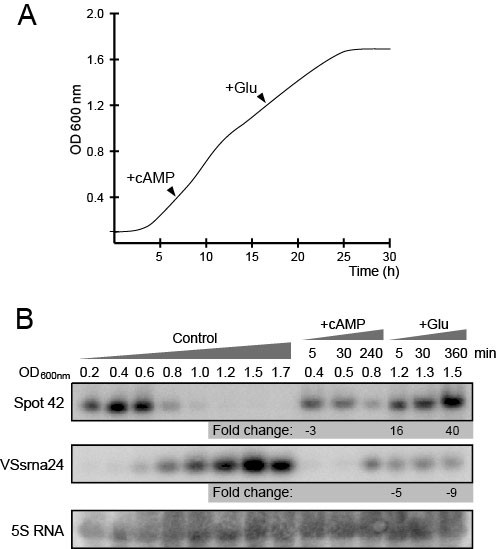
**Monitoring Spot 42 and VSsrna24 expression under different treatments**. (A) *A. salmonicida *was cultured for approximately 30 hours (h) starting at OD_600 nm _0.1, and ending at 1.8 (stationary phase). A typical growth trajectory of *A. salmonicida *in LB is illustrated in the figure. Samples were collected throughout the growth cycle. The culture was split and 1 mM cAMP was added at OD_600 nm _0.4 to one half. Similarly, a second culture was split and 5 mM glucose was added at OD_600 nm _1.2. Untreated cells were used as control. (B) Samples collected in A were subjected to Northern blot analysis. From the control culture samples were collected at OD_600 nm _0.2-1.7, and after the additions of cAMP or glucose samples were collected after 5-240 min (OD_600 nm _0.4-0.8) or 5-360 (OD_600 nm _1.2-1.5) min, respectively. Radio-labeled double-stranded DNA probes were used to monitor the levels of Spot 42 and VSsrna24, and 5S rRNA was used to normalize the result. Fold change values ≥ 2 are shown below samples, and are always comparisons between treated and untreated control samples, at same ODs.

To test if *A. salmonicida *Spot 42 is expressed similar to that in *E. coli*, the bacterium was cultured in LB with 2.5% NaCl added (*A. salmonicida *requires elevated salt concentrations for efficient growth) under standard lab conditions, and samples were collected throughout the growth cycle. At OD_600 nm _0.4 the culture was split, and one half was supplemented with 1 mM cAMP (LB medium contains yeast extract and will contain trace amounts of cAMP, e.g. see Polnisch and Hofmann) [14] (Figure 2A). At early growth phase the intracellular levels of cAMP are expected to be low. Similarly, the starting culture was also split at OD_600 nm _1.2, and glucose was added to one half to a final concentration of 5 mM (i.e., when glucose is expected to be low/exhausted). Cell samples were collected at various time points after the addition of cAMP or glucose to monitor the levels of Spot 42 by Northern blot analysis. Figure 2B shows that Spot 42 is found at high levels during the early phases of growth, but is gradually lost and is below our level of detection at OD_600 nm _1.2. The level of Spot 42 is significantly reduced (3 folds) after addition of cAMP or is significantly increased (16-40 folds) after addition of glucose. These results are in agreement with results from *E. coli*, which suggest that *A. salmonicida *Spot 42 serve roles similar to that in *E. coli*, i.e., in carbohydrate metabolism.

In the same experiment we also monitored the levels of the putative sRNA named VSsrna24, which is encoded from the same IGR as Spot 42. This sRNA produced a signal corresponding to an RNA of approximately 60 nt in length, with an expression pattern opposite to that of Spot 42. Also, expression of VSsrna24 is repressed by glucose (5-9 folds), but appears independent of cAMP. These observations and the close proximity of VSsrna24 to Spot 42 suggested to us that VSsrna24 might also play roles in carbohydrate metabolism, perhaps in concert with Spot 42.

Finally, in a simple experiment we subjected *A. salmonicida *to different stress conditions (i.e., low iron conditions, oxidative stress, low/high temperatures, and alcohol) or to one of two quorum sensing signal molecules (hexanoyl-L-homoserine lactone or N-3-oxo-hexanoyl-L-homoserine lactone), and monitored levels of Spot 42 and VSsrna24 by Northern blot analysis (provided as Additional File 1). This was done to test if expression of Spot 42 and/or VSsrna24 is dependent on external stress factors or communication molecules, which could indicate potential roles in stress response or quorum sensing, respectively. *A. salmonicida *was grown to OD_600 nm _0.5 and subjected to the different treatments. Samples were then collected at various time points after treatment, but none of the stress conditions dramatically changed the expression pattern, maybe except for low iron conditions (addition of 50 uM 2,2'-dipyridyl) that resulted in reduction in Spot 42 levels (2-5 fold).

To summarize, we found that expression of *A. salmonicida *Spot 42 is repressed by cAMP and activated by glucose, whereas expression of a new sRNA, named VSsrna24, is repressed by glucose and is independent of cAMP. We also found that expression of both Spot 42 and VSsrna24 is, in general, not affected by external stress factors or by the addition of quorum sensing signals.

### Microarray analysis of a *A. salmonicida spf *deletion mutant identifies potential roles and targets

We constructed a *spf *deletion mutant (see Methods for details) to identify potential biological roles and possible mRNA targets for *A. salmonicida *Spot 42. The transcriptome of the *spf *deletion mutant was compared on a global scale to that of the wild-type strain by using microarray analysis. To create growth conditions in which Spot 42 was highly expressed in the wild-type strain, the cells were cultured in ASMM minimal medium and then stimulated by adding glucose as the main carbon sources. Specifically, in three independent experiments (biological replicates), cells were grown to OD_600 nm _0.4, and 44.4 mM glucose was added. Samples were collected 15 min after addition of glucose, total RNA was extracted, and the three independent samples were pooled before cDNA synthesis and hybridization to three *A. salmonicida *whole genome custom DNA chips (i.e., *Vibrio salmonicida *V1.0.1 AROS). RNA from glucose treated wild-type and *spf *deletion cells were labeled with fluorescent dyes and run on same DNA chips.

**Figure 3:**
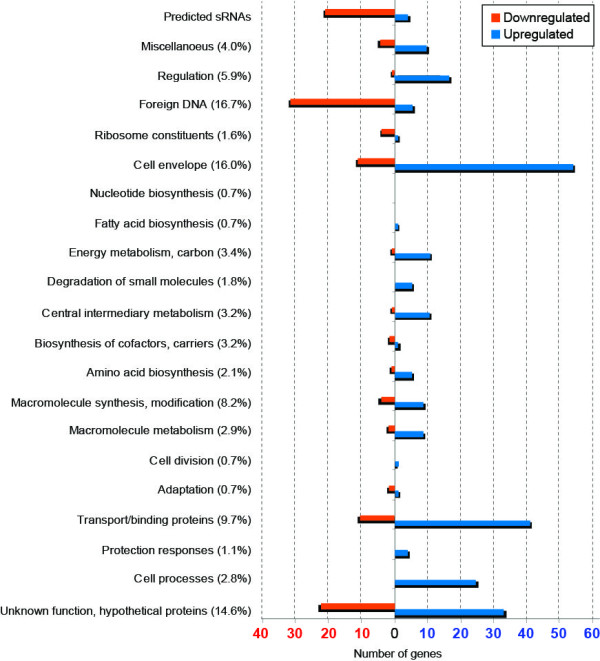
**Overview of microarray results with *spf *deletion mutant compared to the wild-type strain**. Bacteria were grown to OD_600 nm _0.4, then the culture was split and 44.4 mM glucose was added to one half. Cells were collected after 15 min. CDSs with differential gene expression corresponding to up- or downregulation above or below 1.5 fold are divided into functional categories as defined by the Sanger Institute Pathogen Sequencing Unit http://genprotec.mbl.edu/files/MultiFun.html. Numbers in parenthesises indicate in percentage the share of the total number of genes in the genome that each class represents.

Using the LIMMA framework in Bioconductor [15] we considered genes with ≥ 2 fold differential expression and adjusted p-values ≤ 0.05. Figure 3 shows a graphical presentation of the functional classes and the number of differentially up- or downregulated genes in the *spf *deletion mutant 15 min after addition of glucose. Details on differentially regulated genes are shown in Tables 1 and 2 (Additional file 2 contains complete lists of differentially expressed genes). From these data it is clear that the *spf *deletion is affecting the expression of a relatively large number of genes (e.g., 385 genes are ≥ 2 fold differentially expressed after addition of glucose) that belong to a wide range of categories, such as "foreign DNA", "carbohydrate metabolism", "cell envelope", "transport proteins", "motility", "iron homeostasis", and "quorum sensing". Spot 42 therefore has a broad effect on multiple cellular functions.

**Table 1:** Top 30 list of ≥ 2 fold upregulated genes in *spf *deletion mutant after addition of 44.4 mM glucose.

CDS	Gene	Gene product	Log_2 _ratio	Adjusted P-value
VSAL_I1200		putative pirin	3.97	2.49E-04
VSAL_I2193		methyl-accepting chemotaxis protein	2.92	2.97E-04
VSAL_I4139s		VSsrna140 undefined small RNA	2.53	4.98E-04
VSAL_I0799		methyl-accepting chemotaxis protein	2.53	4.02E-04
VSAL_I2318	*flaD*	flagellin subunit D	2.48	4.35E-04
VSAL_II0716		putative exported protein	2.39	3.54E-03
VSAL_I2317	*flaA*	flagellin subunit E	2.33	4.98E-04
VSAL_II0613		putative membrane protein	2.17	8.69E-04
VSAL_I1201		putative IMP dehydrogenase/GMP reductase	2.13	4.44E-04
VSAL_I2319	*flaA*	flagellin subunit C	2.09	5.58E-04
VSAL_I2517	*flaF*	flagellin subunit F	2.07	6.11E-04
VSAL_I2329	*flgM*	flagellar hook-associated protein type 3 FlgM	2.03	4.98E-04
VSAL_I2022	*vcmH*	multidrug efflux pump	1.87	1.20E-03
VSAL_II0587		outer membrane protein, OmpA family	1.87	6.40E-04
VSAL_I2593		gluconate permease	1.81	6.74E-04
VSAL_II0091	*fruK*	1-phosphofructokinase	1.81	9.06E-04
VSAL_II0090	*fruB*	PTS system, fructose-specific IIA/FPR component	1.80	6.11E-04
VSAL_I2578		ABC-type [(GlcNAc)2] transporter, permease protein	1.79	6.11E-04
VSAL_I1857		hypothetical protein	1.78	6.74E-04
VSAL_II0715	*cusB*	putative cation efflux system protein	1.77	8.23E-04
VSAL_I2338	*flgB*	flagellar basal-body rod protein FlgB	1.75	5.13E-04
VSAL_II0231	*cheV*	chemotaxis protein CheV	1.74	8.03E-04
VSAL_I2771	*motX*	sodium-type polar flagellar protein MotX	1.71	7.15E-04
VSAL_II0331		putative exported protein	1.70	6.14E-04
VSAL_I2334	*flgF*	flagellar basal-body rod protein FlgF	1.70	8.55E-04
VSAL_II1080		membrane protein	1.70	7.00E-04
VSAL_I0474	*mshF*	Type IV pilus, mannose-sensitive hemagglutinin protein MshF	1.68	6.11E-04
VSAL_I1401	*tupA*	extracellular tungstate binding protein precursor	1.66	6.86E-04
VSAL_I2327	*fliC*	flagellin subunit A	1.62	7.00E-04
VSAL_II0785		putative exported protein	1.59	6.11E-04
VSAL_I2061		hypothetical protein	1.59	6.11E-04

**Table 2:** Top 30 list of ≥ 2 fold downregulated genes in *spf *deletion mutant after addition of 44.4 mM glucose

CDS	Gene	Gene product	Log_2 _ratio	Adjusted P-value
VSAL_II0612		HTH-type transcriptional regulator, LysR family (pseudogene)	-5.71	1.24E-04
VSAL_I0768		hypothetical protein, putative phage gene	-5.62	1.24E-04
VSAL_I3103s		VSsrna23 small RNA Spot 42	-5.60	1.24E-03
VSAL_I3178s		VSsrna149 tmRNA	-5.19	1.24E-04
VSAL_I1028		gpN major capsid protein	-5.17	1.46E-04
VSAL_p320_13		putative peptidase, S24-like	-5.16	2.49E-04
VSAL_II2002s		VSAsrna3 undefined small RNA	-5.12	1.46E-04
VSAL_I0985		MrdA penicillin-binding protein 2	-5.06	2.49E-04
VSAL_I1029		gpM phage terminase, endonuclease subunit	-5.02	1.24E-04
VSAL_I4069s		VSsrna70 undefined small RNA	-4.72	4.98E-04
VSAL_I0136		IucC siderophore biosynthesis protein	-4.42	1.97E-04
VSAL_I4155s		VSsrna156 undefined small RNA	-4.20	6.14E-04
VSAL_I1027		gpO phage capsid scaffolding protein	-4.10	3.71E-04
VSAL_I3104s		VSsrna24 small RNA	-4.10	2.97E-04
VSAL_I0135		AlcA siderophore biosynthetis protein	-4.04	3.71E-04
VSAL_p54_02		putative mobilization protein	-3.93	3.71E-04
VSAL_p43_01		replication initiation protein	-3.92	1.24E-04
VSAL_I0134		L-2,4-diaminobutyrate decarboxylase	-3.84	2.49E-04
VSAL_I3073r		5S rRNA 5S rRNA undefined product 93740:93859 forward	-3.82	7.53E-04
VSAL_I1039		probable exported protein, putative phage gene	-3.72	2.49E-04
VSAL_I3157t		tRNA transfer RNA-Ser	-3.64	3.13E-04
VSAL_I0137		TonB-dependent iron-siderophore receptor precursor	-3.58	3.08E-04
VSAL_p54_01		acyltransferase	-3.58	4.98E-04
VSAL_I1751		TonB1 TonB protein (pseudogene)	-3.56	3.71E-04
VSAL_I3144t		tRNA tRNA transfer RNA-Leu 842679:842760 reverse	-3.46	8.25E-04
VSAL_I3072r		23S rRNA 23S rRNA undefined product 90756:93646 forward	-3.37	1.04E-03
VSAL_I1040		hypothetical protein, putative phage gene	-3.33	3.13E-04
VSAL_p320_31		putative phage intergrase	-3.32	3.71E-04
VSAL_p43_02		acetyltransferase	-3.25	1.97E-04
VSAL_I3102s		VSsrna22 small RNA RyhB	-3.23	3.08E-04

Major findings are, first, that a gene encoding a pirin protein is by far the most upregulated gene. The log_2 _ratio between *spf *deletion mutant and wild-type is 3.97 (equivalent to ~16 fold increase), whereas the same value for the second most upregulated gene, i.e., VSAL_I2193 that encodes a methyl-accepting chemotaxis protein, is 2.92 (equivalent to ~8 fold increase). The pirin protein (theoretically 283 aa) has 21 and 24% identity, and 45 and 49% similarity to the *E. coli *(231 aa) and *S. marcescens *(313 aa) pirin proteins, respectively, when all positions in the protein sequences are considered (approx. 45% identity in conserved regions). Second, the top 30 list of most upregulated genes is highly enriched with genes involved in motility and chemotaxis [*cheV*, *flaA *(subunit E), *flaA *(subunit C), *flaD*, *flaF*, *flgB*, *flgF*, *flgM*, *fliC*, *motX*, VSAL_I0799 and VSAL_I2193]. Upregulation of genes with functions in motility and chemotaxis is potentially a result of upregulation of the flagellar biosynthesis sigma factor (sigma F or 28), encoded by *fliA *(VSAL_I2290), the spoIIAA anti-sigma F factor antagonist (VSAL_II0328), and finally the anti-sigma 28 factor (VSAL_I2342). These are the most upregulated genes (i.e., 1.7, 1.6 and 1.6 fold, respectively) of all sigma factor-related genes in the complete dataset. Flagella-related genes are known from other bacteria to be regulated by the sigma 28 factor (or its orthologs, e.g., sigma F). Almost all upregulated motility and chemotaxis genes are located in the same gene cluster (approx. VSAL_I2283 to VSAL_I2343). Third, *fruBK *are both on the top 30 list of upregulated genes, whereas *fruA *is also significantly upregulated (2.3 fold upregulated). FruAB constitute the fructose-specific EII-component of the phosphotransferase system (PTS). Therefore, the upregulation of *fruABK *supports a similar role for *A. salmonicida *Spot 42 as in *E. coli *where uptake of less favorable sugars is suppressed by Spot 42 (carbon catabolite repression). Finally, the top 30 list of most downregulated genes is dominated by foreign DNA (phage and plasmid DNA) and genes encoding non-coding RNAs (ncRNAs), including a number of predicted sRNA genes.

In summary, the microarray analysis shows that deletion of *spf *has a broad effect on many genes and cellular functions. Interestingly, the most upregulated gene encodes a putative pirin protein. In *S. marcescens*, pirin has been found to regulate the activity of pyruvat dehydrogenase E1 [16]. Pirin is therefore a key regulator in the central metabolism by selecting the destiny of pyruvat, either through fermentation or respiration through the tricarboxylic acid (TCA) cycle and electron transport. Because *A. salmonicida *Spot 42 directly or indirectly regulates pirin, Spot 42 likely plays a critical regulatory role in the central metabolism.

### Upregulation of pirin and repression of multiple sRNAs are supported by Northern blot analysis

**Figure 4:**
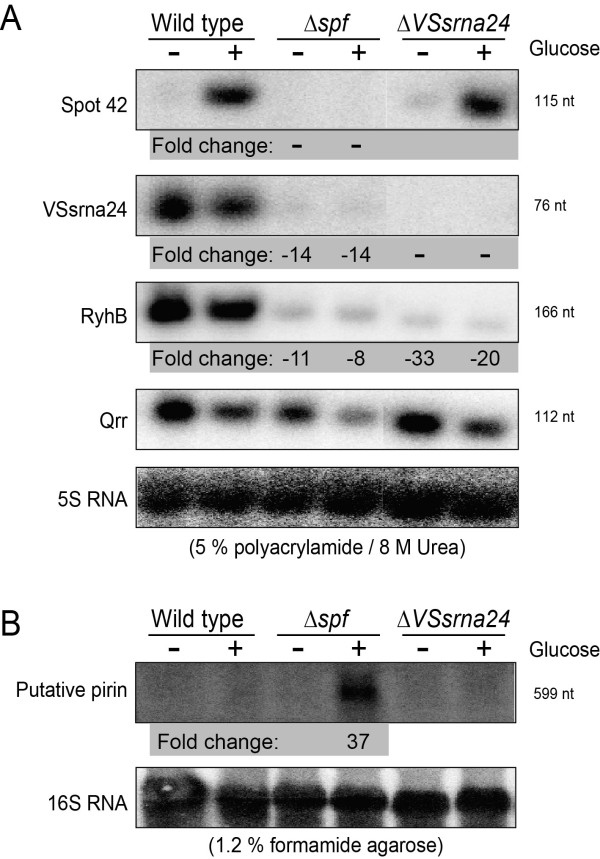
**Validation of selected microarray results with Northern blot analysis**. (A) RNAs from wild-type and *spf *and *VSsrna24 *deletion mutants (Δ*spf *and Δ*VSsrna24*, respectively) were separated on 5% denaturating polyacrylamide gels, transferred to membranes and tested for presence of the sRNAs Spot 42, RyhB, Qrr and VSsrna24. 5S rRNA was used as control and to normalize the results. Plus (+) indicates that glucose was added to the culture 15 min prior to sampling, whereas minus indicates that no glucose was added. Numbers to the right of gel pictures indicate the length of RNAs as measured from the gel. Fold change values ≥ 2 are shown below samples, and are always comparisons between deletion mutants and wild-type control samples, at same conditions. (B) Same samples as described above separated on a 1.2% denaturating formamide gel.

Inspired by findings from the microarray analysis we next used Northern blot analysis to evaluate results of particular interest. First, we wanted to validate levels of Spot 42, and therefore generated a corresponding [α-^32^P]-labeled DNA probe. Figure 4A shows the resulting autoradiogram which confirms that Spot 42 is rich in wild-type cells in the presence of glucose, and is absent from *spf *deletion cells. As expected, Spot 42 is virtually absent in wild-type cells grown without any added glucose.

We were intrigued by the dramatic effect of *spf *deletion on levels of a putative pirin-encoding mRNA. In agreement with the microarray result a pirin probe produced no visible band when using total RNA from wild-type cells, whereas total RNA from *spf *deletion mutant cells treated with glucose produced a distinct and readily visible band of the expected size (Figure 4B). The increase in intensity corresponds to a fold change value of approx. 37 folds (approx. 16 folds in microarray analysis). We next used the RNAhybrid software [17] to test for potential interactions between Spot 42 and the 5' region of the pirin mRNA. This software calculates the most energetically favorable base pairing between a small RNA and a larger target RNA [17]. Additional file 3 shows that when using *A. salmonicida *Spot 42 (excluding the terminator) and a sequence extending from -100 nt to + 50 relative to the pirin start codon as input sequences to the program, significant potential for base-pairing between Spot 42 and the 5' untranslated region (UTR) of the pirin mRNA is found. Given that Spot 42 binds directly to this region then this would be similar to the most common form of sRNA-mRNA interaction, i.e., base pairing within the 5' UTR region [1]. Further analysis to address potential interactions between Spot 42 and pirin mRNA will come from biochemical methods, such as gel mobility shift assay, fusion-reporter assays, structure probing and site directed mutagenesis.

Our microarray data suggest that many predicted sRNAs are significantly downregulated in the *spf *deletion mutant. This can potentially be explain by reduction in Hfq (encoded by *hfq*) and/or upregulation of RNaseE (encoded by *rne*), which can destabilize sRNAs in general. However, the microarray data indicate that mRNA levels of the corresponding mRNAs are virtually unchanged. Next, we designed probes against the new VSsrna24, and the RyhB and Qrr sRNAs that are critical components in regulation of iron homeostasis and quorum sensing, respectively. These sRNAs were found to be downregulated approx. 14, 10 and 2 folds, which is in good agreement with the corresponding numbers (approx. 17, 10 and 4 folds, respectively) from the microarray analysis. The underlying mechanism(s) for the apparent reduction in sRNA levels remains however unclear. It should however be noted that VSsrna24 is encoded 219 nt downstream of the deleted region in the *spf *mutant strain, and it is therefore possible that the deletion has affected the promoter and therefore expression of VSsrna24. In the same experiment we also included a *VSsrna24 *deletion mutant. Here, RyhB is also significantly downregulated (19/35 folds with/without glucose), whereas levels of Spot 42 and Qrr appear unchanged.

## Conclusions

**Figure 5:**
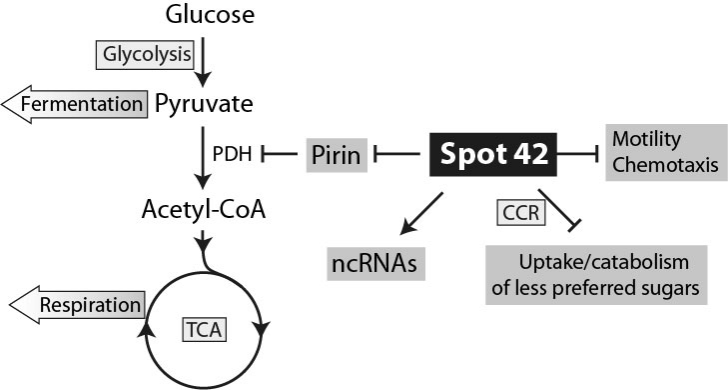
**A model for Spot 42 regulation in *A. salmonicida***. The model is based on microarray and Northern blot analyses from this study, which suggest that Spot 42 downregulates Pirin (key regulator of puruvat dehydrogenase complex in central metabolism), genes for uptake/catabolism of less preferred sugars in a carbon catabolite repression (CRR)-like manner, and genes involved in motility and chemotaxis. The results also suggest that Spot 42 activates expression of other ncRNAs (e.g., the sRNA RyhB).

We have studied potential biological roles of a *A. salmonicida *Spot 42 homolog and discovered a neighboring sRNA, named VSsrna24. The expression of Spot 42 is similar to that in *E. coli*, i.e., expression is repressed by cAMP and activated by glucose. Expression of VSsrna24 is, in contrast, repressed by glucose but unaffected by cAMP. A microarray analysis revealed that deletion of *spf *affects a wide range of cellular processes and the expression of a surprisingly large number of genes. After addition of glucose at mid exponential growth phase (i.e., when Spot 42 is highly expressed in the wild-type strain), the most differentially upregulated gene in the *spf *deletion mutant was VSAL_I1200, which encodes a pirin protein with 21% identity and 45% similarity to the *E. coli *pirin. Other notable results are upregulation of many genes involved in motility and chemotaxis, and downregulation of predicted sRNA genes and foreign DNAs. Expression of Spot 42, VSsrna24, Qrr, RyhB and pirin were validated by Northern blot analysis. Figure 5 shows a hypothesis of the main findings. It should be stressed that it should be regarded as a hypothesis that must be tested further, and solid evidence can come from e.g., gel mobility shift assay or fusion-reporter assays to address potential interactions between Spot 42 and potential mRNA targets, or complementation assay to restore Spot 42 functions.

The majority of bacterial regulatory RNAs, like sRNAs, probably remain to be identified. Eighty-seven sRNAs are known in *E. coli *[2], whereas the corresponding number is much lower for other bacteria. Once identified, finding the function and targets of each sRNA represents a formidable task. In general, the current knowledge on sRNA function is therefore limited. Although one or a few targets have been identified for a given sRNA, it is likely that many additional targets remain to be found. For example, in *E. coli*, the transcript of the multi-cistronic *gal *operon was for many years the only known Spot 42 target [10], but a recent microarray analysis in which over-expression of *spf *with an isopropyl β-D-thiogalactopyranoside (IPTG)-inducible promoter was used, suggests many additional targets [11]. The list of targets is enriched in genes involved in sugar transport (*dppB*, *IldP*, *nanC*, *nanT*, *srlA *and *xylF*) or sugar catabolism (*ebgC*, *fucI*, *fucK *and *galK*). We have used a knock-out approach to find overexpressed genes that could be potential Spot 42 targets in *A. salmonicida*. Interestingly, the majority of genes that corresponds to mRNA targets in *E. coli *are apparently not present (i.e., they produce no significant hits in BLASTP searches) in the *A. salmonicida *genome (i.e., *IldP*, *nanC*, *srlA*, *ebgC*, *fucI*, *galK *and *gsp*). Also, apparent homologs of the remaining *E. coli *targets were in general not significantly differentially expressed in our dataset. These results suggest that there are significant differences in Spot 42 regulation between *E. coli *and *A. salmonicida*.

Perhaps our most intriguing finding is that *A. salmonicida *Spot 42 appears to directly or indirectly regulate the levels of pirin mRNA. This is in agreement with its currently known role in carbohydrate metabolism, and that the level of Spot 42 is repressed by cAMP and activated by glucose. We are also pursuing potential roles of the neighboring sRNA, named VSsrna24. Its expression is repressed by glucose, and we suspect that this sRNA also could have important roles in carbohydrate metabolism.

## Methods

### Bacterial strains and growth

*A. salmonicida *LFI1238 and the corresponding *spf *deletion mutant were cultured in LB medium with 2.5% NaCl added, or in the *A. salmonicida*-specific minimal media ASMM, at 12-16°C in a shaking incubator at 200-230 rpm. ASMM contains the following compounds in mM: NaCl, 256.7; KCl, 9.4; MgCl_2 _· 6H_2_O, 50.075; FeCl_3 _· 6H_2_O, 0.0126; NH_4_Cl, 18.7, K_2_HPO_4_, 0.58; Trizma base, 41.3; CaCl2 · 2H_2_O, 0.225; ZnCl_2_, 0.0085, CoCl_2 _· 6H_2_O, 0.0055; CuSO_4 _· 5 H_2_O, 0.005; Na_2_MoO_4_, 0.007; MnCl_4 _· 2H_2_O, 0.011; cycteine, 0.5; isoleucine, 0.5; methionine, 0.5; valine, 2.0; glutamic acid, 40; glucose or galactose (added when indicated), 44.4 mM.

*A. salmonicida *was subjected to multiple treatments by first growing the bacterium in LB medium with 2.5% NaCl at 16°C to mid-exponential growth phase (OD_600 nm _~0.6). Cultures were then divided into nine equally sized cultures, one for each stimulation, i.e., 50 μM 2,2'-dipyridyl iron chelator, 1 mM hydrogen peroxide, 500 μM paraquat (N, N'-dimethyl-4,4'-bipyridinium dichloride), 0.5% ethanol, 2 μg/ml hexanoyl-L-homoserine lactone (HHL), 2 μg/ml N-3-oxo-hexanoyl-L-homoserine lactone (OHHL), 4°C or 20°C growth temperatures, and one non-stimulated control culture. Ten ml samples were collected 0, 2, 5, 10 and 30 min after stimulation. Cells were harvested by centrifugation at 3,500 × g for 10 min. Pelleted cells were then snap-frozen in a combination of dry-ice and ethanol, and kept at -70°C prior to RNA isolation.

For cAMP and glucose stimulations *A. salmonicida *was cultured in LB medium at 12°C and split two times during growth. At OD_600 nm _0.4, the culture was split and 1 mM cAMP was added to one half and grown under the same conditions as the untreated control for 240 min. Ten ml samples were collected 5, 30 and 240 min after stimulation. Similarly, at OD_600 nm _1.2, the untreated culture was again split and one half was stimulated with 5 mM D(+)-glucose and grown under the same conditions as the untreated control culture for 360 min. Ten ml samples were collected 5, 30 and 360 min after stimulation.

Samples used for Northern blot and microarray analyses were prepared by culturing *A. salmonicida *wild-type and a *spf *deletion mutant strains at 12°C in ASMM without any added carbon source to OD_600 nm _0.4. At this density the culture was split into two halves. Glucose to 44.4 mM was added to one half and the other was used as the untreated control. A 15 ml sample was collected 15 min after stimulation and harvested as described above.

### Construction of *Spf *and *VSsrna24 *deletion strains

The construction of *Spf *and *VSsrna24 *deletion strains was performed essentially as previously described [18]. A suicide vector (pDM4) containing regions flanking the *spf *or *VSsrna24 *genes were used. The flanking regions were joined by overlap PCR, i.e., two PCR products containing the flanking regions with overlapping ends are extended and then used as template with the outermost primers from the first two PCRs. The final PCR product was cloned into the pDM4 plasmid and introduced into *A. salmonicida *by conjugal mating with the *E. coli *S17-1 λ pir donor strain. Transconjugants were selected by plating the conjugation mix on LB plates containing 2 μg/ml chloramphenicol at 12°C. *E. coli *grows very poorly at 12°C and was therefore selected against. Colonies were next transferred to LB plates with 5% sucrose to induce expression of the *sacB *suicide gene. The product is lethal to Gram-negative bacteria [19]. This step selects against the plasmid, and integrated plasmids (they are most likely to have integrated into chromosomal locations similar to those that were introduced into the plasmid) must therefore be removed from chromosomal positions in a recombination event in order for the bacterium to survive. Resulting chloramphenicol-sensitive colonies were analyzed by PCR to screen for bacteria with the desired chromosomal deletion. PCR products of expected sizes were sequenced to confirm the deletion.

### 5' rapid amplification of cDNA ends (RACE) for mapping of the 5' end

5' RACE assays were carried out essentially as previously described [20] using the GeneRacer kit (Invitrogen), 6 μg of total RNA, and a Spot 42 or VSsrna24-specific oligonucleotide (5'-GCCAAATCCAATAACGTGAAAC-3' or 5'-GCTAAGGTGGGGAAATGG-3', respectively).

### Microarray analyses

Total RNA was extracted from cells using the RNAisol reagent (5 PRIME) followed by a subsequent DNA removing step with DNAfree kit (Applied Biosystems). Salts and leftover traces of DNA were removed by RNeasy Minelute Cleanup kit (Qiagen). Quality of extracted RNA was examined by NanoDrop, and presence of RNase or DNase activity was checked by incubating the RNA with 500 ng plasmid DNA for 1 h at 37°C, followed by inspection of degradation on a EtBr-stained 1% agarose gel. cDNA was constructed from 15 μg purified RNA using Aminoallyl cDNA labeling kit (Applied Biosystems) and CyDye™ Post-Labeling Reactive Dye Pack (GE Healthcare). The labelled samples were hybridized to "*Vibrio salmonicida *V1.0.1 AROS" slides at 42°C on a TECAN HS4800 hybridisation station. Following the hybridizations, the slides were washed once in 0.1 × SSC/0.1% SDS for 5 min at 42°C, once in 0.1 × SSC/0.1% SDS for 10 min at room temperature, and finally four times in 0.1 × SSC for 1 min at room temperature. The slides were run in triplets, including one dye swap per triplet. Slides were scanned using a GenePix 4000B scanner (Axon Instruments Inc.) and GenePix Pro v6.1 software. The expression data were analysed using the LIMMA framework in Bioconductor http://www.bioconductor.org. Microarray data is available at the NCBI Gene Expression Omnibus (GEO) database with the GSE28087 as accession number.

### Northern blot analyses

Total RNA was isolated from bacterial cultures using the TRIzol RNA isolation procedure (Invitrogen) and quantified by spectrophotometric methods. Northern blot analysis was done as previously described [12]. Briefly, 10 μg total RNA was separated on 1.2% formamide agarose gels or 8 M urea/5% polyacrylamide gels, and then transferred to a nylon membrane by upward capillary transfer or electroblotting, respectively. RNA species were detected on membranes using [α-^32^P]dCTP-labeled double-stranded DNA probes (PCR products), and signals were collected on phosphoimaging screens (Fujifilm) and scanned on a BAS-5000 phosphoimager (Fujifilm). ImageGauge v4.0 (Fujifilm) was used to measure the strength of signals, and 5S ribosomal RNA was used to normalize the resulting values.

### Promoter and TFBS prediction

The region immediately upstream of *spf *was screened for CRP binding sites. A consensus sequence, made from 211 CRP binding sites (5'-WWWTGTGATCTRBRTCACAWWT-3') from RegulonDB [21], was used along with the pattern search algorithm Fuzznuc (part of EMBOSS software analysis package) [22]. A maximum of 6-9 mismatches of the 22 bp consensus were allowed. This predicted four overlapping sites stretching 44 nt in length. The CRP binding site (5'-TTTTGTGATGGCTATTAGAAAT-3') upstream of the *E. coli spf *gene [7,8] has 5 mismatches to this consensus. *E.coli *and *A. salmonicida *CRP binding sites share 12 conserved nt. In addition, the MEME [23] and Consensus [24] software tools were also used to search for any additional conserved sequence motifs in the promoter region of the vibrio Spot42 sequences. Both methods predicted a 42 nt conserved site overlapping the CRP site predicted from the known consensus. However, there is weak conservation among the predicted CRP sites in vibrios. The BPROM program (from http://www.softberry.com) was used to predict -10 and -35 promoter sequences.

### Computational prediction of Spot42-pirin mRNA base-pairing

The program RNAhybrid [17] was used to identify potential base-pair interactions between Spot 42 and the 5' UTR region of the pirin mRNA (VSAL_I1200). The search was restricted to mRNA sequences located 100 nt upstream and 50 nt downstream of the start codon, with default program settings.

## Authors' contributions

GÅH participated in the design of experiments, performed experiments and participated in drafting the manuscript. RA participated in design of experiments and helped draft the manuscript. Bioinformatics analyses of microarray data were done by CGF and EH. NPW and PH conceived the study and cosupervised the work. All authors read and approved the final manuscript.

## Supplementary Material

Additional file 1 **Monitoring Spot 42 and VSsrna24 expression under different treatments or stress conditions with Northern blot analysis**. *A. salmonicida *was grown to mid exponential phase (OD600_nm 0.5_) and split into nine smaller cultures. These were subjected to hexanoyl-L-homoserine lactone (HHL; 2 μg/ml), N-3-oxo-hexanoyl-L-homoserine lactone, (OHHL; 2 μg/ml), N, N'-dimethyl-4,4'-bipyridinium dichloride, (paraquat; 500 μM), hydrogen peroxide (H_2_O_2_; 1 mM), iron-chelator (2,2-dipyridyl; 50 μM), ethanol (EtOH; 0,5%), 4°C or 20°C, or used as control (16°C). Samples were harvested between 0-30 min, and after 8 and 20 hours (h) for the control culture. RNA samples were run on two gels and transferred to two membranes (Membranes 1 and 2) for practical reasons, and subjected to Northern blot analysis. Radio-labeled double-stranded DNA probes were used to monitor the levels of Spot 42 and VSsrna24, and 5S rRNA was used to normalize the result. None of the conditions resulted in significant changes in the expression pattern, except after addition of 50 uM 2,2'-dipyridyl which produced 2-5 fold reduction in Spot 42 levels. Click here for file

Additional file 2 **Complete lists of differentially expressed genes**. Microarray data from *A. salmonicida spf *deletion mutant grown in ASSM minimal medium and stimulated by added glucose. Click here for file

Additional file 3 **Potential for base-pairing between Spot 42 and pirin mRNA**. The RNAhybrid software was used to calculate the most energetically favorable base pairing between *A. salmonicida *Spot 42 (excluding the terminator stem) and the 5' region of the pirin mRNA (-100 nt to + 50 relative to the pirin start codon). Click here for file
