# Supplementary figures and images for: Expression profiling reveals Spot 42 small RNA as a key regulator in the central metabolism of Aliivibrio salmonicida
Source: BMC Genomics. 2012 Jan 24;13:37. doi: 10.1186/1471-2164-13-37 (PMC3295665; doi:10.1186/1471-2164-13-37)

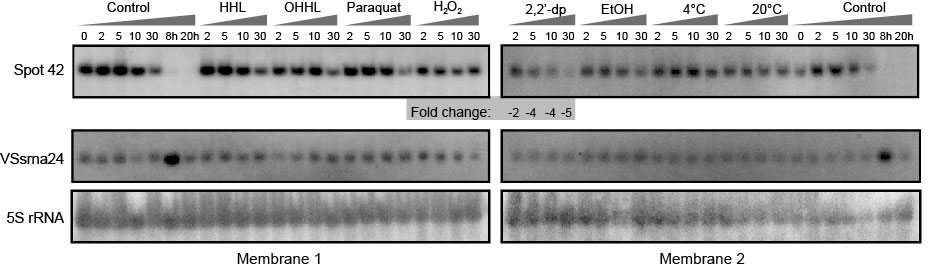

Supplement: Additional file 1 — Monitoring Spot 42 and VSsrna24 expression under different treatments or stress conditions with Northern blot analysis. A. salmonicida was grown to mid exponential phase (OD600nm 0.5) and split into nine smaller cultures. These were subjected to hexanoyl-L-homoserine lactone (HHL; 2 μg/ml), N-3-oxo-hexanoyl-L-homoserine lactone, (OHHL; 2 μg/ml), N, N'-dimethyl-4,4'-bipyridinium dichloride, (paraquat; 500 μM), hydrogen peroxide (H2O2; 1 mM), iron-chelator (2,2-dipyridyl; 50 μM), ethanol (EtOH; 0,5%), 4°C or 20°C, or used as control (16°C). Samples were harvested between 0-30 min, and after 8 and 20 hours (h) for the control culture. RNA samples were run on two gels and transferred to two membranes (Membranes 1 and 2) for practical reasons, and subjected to Northern blot analysis. Radio-labeled double-stranded DNA probes were used to monitor the levels of Spot 42 and VSsrna24, and 5S rRNA was used to normalize the result. None of the conditions resulted in significant changes in the expression pattern, except after addition of 50 uM 2,2'-dipyridyl which produced 2-5 fold reduction in Spot 42 levels. [file 1471-2164-13-37-S1.JPEG]

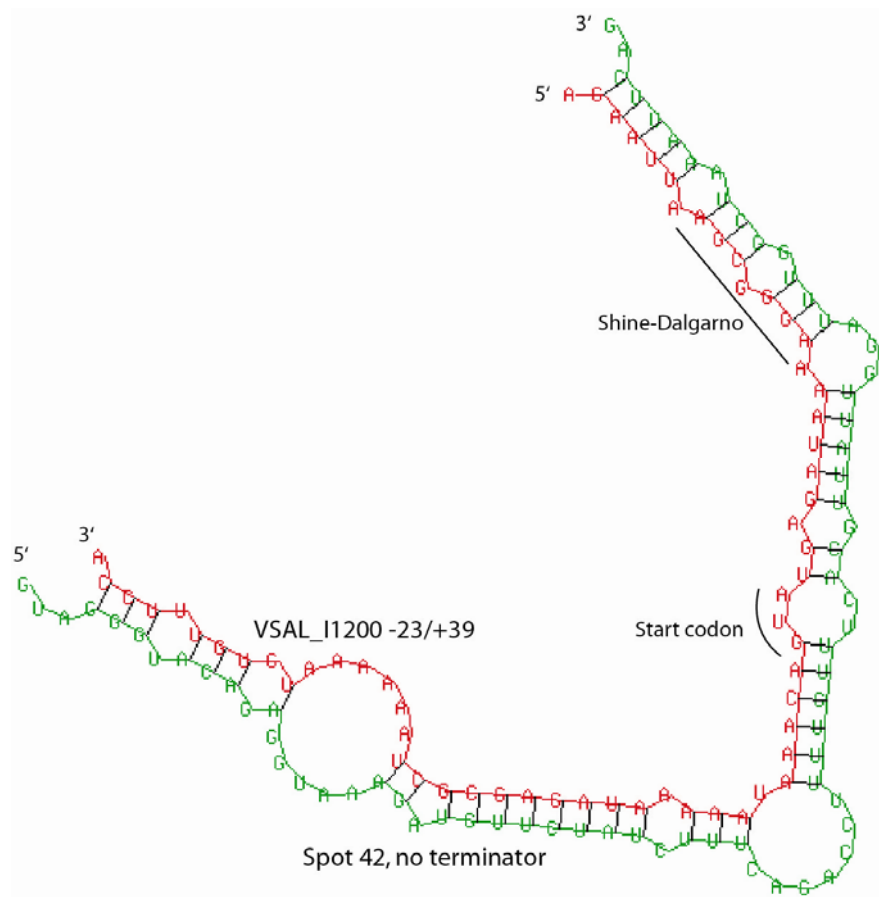

Supplement: Additional file 3 — Potential for base-pairing between Spot 42 and pirin mRNA. The RNAhybrid software was used to calculate the most energetically favorable base pairing between A. salmonicida Spot 42 (excluding the terminator stem) and the 5' region of the pirin mRNA (-100 nt to + 50 relative to the pirin start codon). [file 1471-2164-13-37-S3.PDF]
